# Diarrheagenic pathogens in adults attending a hospital in Singapore

**DOI:** 10.1186/s12879-016-1354-0

**Published:** 2016-01-28

**Authors:** Man Ling Chau, Sri Harminda Pahm Hartantyo, Min Yap, Joanne Su Lin Kang, Kyaw Thu Aung, Ramona Alikiiteaga Gutiérrez, Lee Ching Ng, Clarence C. Tam, Timothy Barkham

**Affiliations:** 1 Environmental Health Institute, National Environment Agency, 11 Biopolis Way, #04-03/04, Helios Block, Singapore, 138667 Singapore; 2 Saw Swee Hock School of Public Health, National University of Singapore, Tahir Foundation Building, 12 Science Drive 2, #10-01, Singapore, 117549 Singapore; 3 London School of Hygiene & Tropical Medicine, Keppel Street, London, WC1E 7HT UK; 4 Department of Laboratory Medicine, Tan Tock Seng Hospital, 11 Jalan Tan Tock Seng, Singapore, 308433 Singapore

**Keywords:** Diarrhoea, Pathogens, Adult hospital patients, Singapore, Acute gastroenteritis, Foodborne

## Abstract

**Background:**

Singapore’s diarrhoeal notification system is based on specific pathogens. Official data may thus be skewed towards notifiable diseases. Limited information is available on the profiles of aetiological agents responsible for acute gastroenteritis (AGE) cases, especially among the adult population. To understand the frequency and distribution of potential causative agents of diarrheal disease in Singapore, we screened adults’ stool samples collected from a large public hospital.

**Methods:**

The stool samples were screened for 18 diarrheagenic pathogens using a combination of commercial multiplex polymerase chain reaction (PCR), in-house singleplex PCR and immunochromatographic assays. One hundred adult faecal samples that were collected from October 2013 to January 2014 for routine diagnostic purposes and submitted for culture at Tan Tock Seng Hospital, Singapore were used.

**Results:**

Pathogens were detected in 32 % of the samples. The predominant organisms encountered were norovirus genogroup II (11 %), *Aeromonas* spp. (9 %) and *Campylobacter* spp. (5 %). One sample was positive for both verocytotoxigenic *E. coli* (VTEC) and *E. coli* O157:H7. Two other samples were positive for VTEC only, and one other sample was positive for *E. coli* O157:H7 only. Astrovirus, *C. perfringens, Shigella* spp. and toxigenic *C. difficile* were each detected in 2 % of the samples. *Cryptosporidium parvum, Giardia lamblia,* group A rotavirus, *Salmonella* spp. and *Vibrio* spp. were each detected in 1 % of the samples. No *L. monocytogenes, Y. enterocolitica,* enteric adenovirus, or norovirus genogroup I were detected.

**Conclusion:**

Our preliminary findings suggest that pathogens causing non-notifiable diseases might have contributed considerably to the adult hospitalised AGE cases. However, as the samples were from an adult hospital, the data obtained may not be representative of the whole community. Thus, a larger study to collect clinical samples and risk exposure data from primary healthcare clinics and children hospital is planned for, to gain a more holistic perspective on the epidemiology of AGE in Singapore. A larger study may also offer valuable insights for improving the approach of microbiological surveillance of food, as well as strategizing inspection efforts along the food supply chain by public health authorities.

## Background

Acute gastroenteritis (AGE) is caused by a wide range of enteric bacteria*,* viruses, protozoa and helminths [1]. Although mortality from AGE is lower in high-income countries, morbidity is still substantial [1, 2]. It is estimated that 15 % of the population in the United States and 27 % of the population in the United Kingdom are affected by AGE per year [3, 4]. In Singapore, the incidence of polyclinic attendances due to acute diarrhoea was 2.2 % of the population in 2009 compared to 2.5 % of the population in 2013 [5, 6]. National surveillance statistics show that the incidence of food poisoning outbreaks (two or more cases epidemiologically linked to a common source) was 4.0 per 100,000 population in 2009 compared to 4.6 per 100,000 population in 2013 [5, 6]. These reported numbers are likely to be an under-estimation of the disease burden, as general acute gastroenteritis (AGE) is not a legally notifiable disease in Singapore, with the exception of specific diseases such as salmonellosis, campylobacteriosis, cholera, enteric fever and hepatitis [7].

Given the lack of information on aetiological agents causing AGE in Singaporean adults, a preliminary study was conducted to determine the frequency and distribution of diarrheagenic pathogens in adults’ residual stool samples collected from a hospital in Singapore. This hospital was chosen as it is one of the largest public hospitals in Singapore [8]. Between March 2014 and February 2015, it had a total number of 2,596 admissions due to gastroenteritis, of which approximately 40 % of the gastroenteritis cases were with complications [9, 10]. It is aimed that through this pilot study, preliminary data can be obtained to guide us in the design of a potentially larger multi-site study to understand the epidemiology of AGE in Singapore.

## Methods

### Specimens

One hundred anonymised faecal samples from adult patients in Tan Tock Seng Hospital, between October 2013 and January 2014, were used. These were residual samples leftover from samples submitted as part of routine adult patient investigation. Only samples submitted within 3 days of admission were used to exclude samples from nosocomial diarrheal cases. No further clinical data were obtained. Samples were kept frozen at -80 °C and transported on ice to the laboratory at the Environmental Health Institute (EHI) of the National Environment Agency (NEA), Singapore. Samples were then stored at -80 °C until laboratory analysis. Ethical approval was granted by the Institutional Review Board of the National Healthcare Group, Singapore. Samples were analysed for the presence of 18 diarrheagenic pathogens using a combination of nucleic acid tests and immunochromatographic assays, as illustrated in Table 1.

**Table 1:** Positivity rate (%) of targeted organisms detected in faecal samples (*n* = 100) by respective screening methods

Organism(s)	Screening method	% positivity
Norovirus GII	Seeplex diarrhoea ACE system	11
*Aeromonas* spp.	Seeplex diarrhoea ACE system, In-house singleplex PCR system	9^a^
*Campylobacter* spp.	Seeplex diarrhoea ACE system, In-house singleplex PCR system	5^b^
Astrovirus	Seeplex diarrhoea ACE system	2
*C. perfringens*	Seeplex diarrhoea ACE system	2
*Shigella* spp.	Seeplex diarrhoea ACE system	2
Toxigenic *C. difficile*	Seeplex diarrhoea ACE system, RIDA QUICK *C. difficile* GDH kit, RIDA QUICK *C. difficile* toxin A/B kit	2^c^
Verocytotoxigenic *E. coli* (VTEC)	Seeplex diarrhoea ACE system	2
*Cryptosporidium parvum*	RIDA QUICK *Cryptosporidium/ Giardia* combi kit	1
*E. coli* O157:H7	Seeplex diarrhoea ACE system	1
*Giardia lamblia*	RIDA QUICK *Cryptosporidium/ Giardia* combi kit	1
Group A rotavirus	Seeplex diarrhoea ACE system	1
*Salmonella* spp.	Seeplex diarrhoea ACE system, In-house singleplex PCR system	1^d^
*Vibrio* spp.	Seeplex diarrhoea ACE system	1
VTEC and *E. coli* O157:H7	Seeplex diarrhoea ACE system	1
Enteric adenovirus	Seeplex diarrhoea ACE system	0
*L. monocytogenes*	In-house singleplex PCR	0
Norovirus GI	Seeplex diarrhoea ACE system	0
*Y. enterocolitica*	Seeplex diarrhoea ACE system	0

^a^Nine samples were detected positive for *Aeromonas* spp. using Seeplex diarrhoea ACE system but five were detected positive by in-house singleplex PCR

^b^Five samples were detected positive for *Campylobacter* spp. using in-house singleplex PCR but none were detected positive by Seeplex diarrhoea ACE system

^c^Two samples were detected positive for both *Clostridium* GDH and *Clostridium* toxin A/B using RIDA Quick lateral flow kits, and for *Clostridium* toxin B using Seeplex diarrhoea ACE system

^d^One sample was detected positive for *Salmonella* spp. by both in-house singleplex PCR and Seeplex diarrhoea ACE system

### Nucleic acid tests

A 10 % suspension of each faecal sample was prepared using Butterfield’s Phosphate Buffer (3 M, Minnesota). Three-hundred microlitres of each 10 % faecal suspension were heated at 100 °C for 10 min. Upon cooling to ambient temperature, the suspension was centrifuged for 10 min at 13,000 rpm and 200 μl of the supernatant were obtained for simultaneous extraction of bacterial and viral nucleic acids using Ribo_spin vRD kit (Geneall, Korea) according to the manufacturer’s instructions. First strand cDNA was synthesized using Maxima H Minus First Strand cDNA synthesis kit (ThermoScientific, Lithuania) using a random hexamer primer according to the manufacturer’s instructions. Nucleic acids obtained were subsequently used for the screening of *Aeromonas* spp., *Campylobacter* spp., *Clostridium difficile* toxin B gene, *Clostridium perfringens, Shigella* spp., verocytotoxigenic *Escherichia coli, Escherichia coli* O157:H7, *Salmonella* spp., *Yersinia enterocolitica, Vibrio* spp., astrovirus*,* group A rotavirus, norovirus genogroup I, norovirus genotype II and enteric adenovirus using Seeplex Diarrhoea ACE system (Seegene, Korea) according to the manufacturer’s instructions. The same samples were also screened for *Aeromonas* spp., *Campylobacter* spp., *Salmonella* spp. and *Listeria monocytogenes* using in-house singleplex PCR assays with published primers (Table 2). In each singleplex PCR, 5 μl of the sample DNA were added to a master-mix consisting of 1X Q5 reaction buffer (New England Biolabs, Massachusetts), 200 μM of dNTPs (1^st^ BASE, Singapore), 0.5 μM of the respective forward and reverse primer (Integrated DNA Technologies, Singapore), 1U of Q5 Hot Start High Fidelity DNA polymerase (New England Biolabs, Massachusetts) and nuclease free water to attain a final volume of 50 μl. For the screening of *Aeromonas* spp, *Campylobacter* spp. and *Salmonella* spp., amplification was conducted in a thermal cycler (ABI Systems GeneAmp PCR system 9700) with the following temperature ramping: 98 °C for 30 s, followed by 35 cycles of 98 °C for 10 s, 62 °C for 30 s, 72 °C for 30 s, and finally 72 °C for 10 min. For the screening of *L. monocytogenes,* the following temperature ramping was performed: 98 °C for 30 s, followed by 35 cycles of 98 °C for 10 s, 61 °C for 30 s, 72 °C for 30 s, and finally 72 °C for 10 min. All PCR amplified product sizes were analysed using QIAxcel DNA screening kit (Qiagen, Hilden). Sequencing of the singleplex PCR products was performed by a commercial company (AITbiotech Pte Ltd, Singapore) using BigDye Terminator Cycle Sequencing kit (Applied Biosystems, USA). Nucleotide sequences were then aligned using SeqMan Pro software (DNASTAR, US) and compared against the online BLAST database (http://blast.st-va.ncbi.nlm.nih.gov/Blast.cgi) to confirm the presence of the targeted pathogens.

**Table 2:** Primers used for the detection of *Campylobacter* spp., *Salmonella* spp. and *Listeria monocytogenes,* as well as the detection and characterisation of *Vibrio cholerae*

Parameter	Target gene	Forward primer (5′-3′)	Reverse primers (5′-3′)	Concentration (μM)	Size (bp)	References
*Aeromonas* spp.	*16 s rDNA*	GGGAGTGCCTTCGGGAATCAGA	TCACCGCAACATTCTGATTTG	0.5	356	[14]
*Campylobacter* spp.	*16 s rDNA*	GGTGTAGGATGAGACTATATA	TTCCATCTGCCTCTCCCY	0.5	439	[54]
*Salmonella* spp.	*invA*	ACAGTGCTCGTTTACGACCTGAAT	AGACGACTGGTACTGATCGATAAT	0.5	244	[55]
*Listeria monocytogenes*	*InlA*	ACGAGTAACGGGACAAATGC	CCCGACAGTGGTGCTAGATT	0.5	800	[56]
*V. cholerae*	*toxR*	GAAGCTGCTCATGACATC	AAGATCAGGGTGGTTATTC	0.01	275	[57]
*V. cholerae* serogroup O1	*Wbe (O1)*	GTTTCACTGAACAGATGGG	GGTCATCTGTAAGTACAAC	0.05	192	[15]
*V. cholerae* biotype (El Tor)	*tcpA*	CACGATAAGAAAACCGGTCAAGAG	CGAAAGCACCTTCTTTCACGTTG	0.05	451	[58]
*V. cholerae* virulence genes	*ctxA*	ACAGAGTGAGTACTTTGACC	ATACCATCCATATATTTGGGAG	0.05	308	[15]
	*ctxB*	ATGAGGCGTTTTATTATTCCATACAC	TACCAGGTAGTCAACATATAGATTCA	0.05	128	[59]

### Immunochromatography assays

A 50 % suspension of each faecal sample was prepared using Butterfield’s Phosphate Buffer (3 M, Minnesota). Suspensions were screened for the presence of *Cryptosporidium parvum* and *Giardia lamblia* using RIDA QUICK *Cryptosporidium/ Giardia* Combi kit (R-Biopharm AG, Germany), as well as *Clostridium difficile* glutamate dehydrogenase (GDH) using RIDA QUICK *Clostridium difficile* GDH kit (R-Biopharm AG, Germany) according to the manufacturer’s instructions. xSamples which were positive for *Clostridium difficile* GDH underwent further screening for toxins using RIDA QUICK *Clostridium difficile* toxin A/B kit (R-Biopharm AG, Germany) according to the manufacturer’s instructions.

### Isolation and characterisation of pathogens

Only one *Salmonella* and one *Vibrio cholerae* isolate were successfully recovered from PCR-positive samples. The isolation of *Aeromonas* spp*, Shigella* spp. and verocytotoxigenic *E. coli* (VTEC) were unsuccessful. Their isolation and characterisation procedures are as described below.

### Isolation and characterisation of *Salmonella* spp.

One gram of each faecal sample that was tested positive for *Salmonella* spp. using Seeplex Diarrhoea ACE system was suspended into 9 ml of the Universal Pre-enrichment Broth (Acumedia, Michigan) and incubated at 37 °C for 24 h. One millilitre of the enriched broth was then sub-cultured into 9 ml of the 2X Rappaport-Vassiliadis Enrichment Broth (Neogen, Michigan) and incubated at 42 °C for 24 h. After incubation, a 10 μl loopful of the enriched broth was sub-cultured onto Hektoen Enteric Agar (Oxoid, Hampshire). Presumptive green colonies with black centres on Hektoen Enteric Agar were confirmed using API 20E strips (bioMérieux, France). Serological groups of the isolates were determined using Wellcolex Colour *Salmonella* latex agglutination tests according to the manufacturer’s instructions (Remel Europe, UK).

### Isolation and characterisation of *Vibrio cholerae*

One gram of each faecal sample that tested positive for *Vibrio* spp. using Seeplex Diarrhoea ACE system was suspended into 9 ml of the Universal Pre-enrichment Broth (Acumedia, Michigan) and incubated at 37 °C for 24 h. After incubation, a 10 μl loopful of the enriched broth was streaked onto Thiosulfate-Citrate-Bile Salt-Sucrose (TCBS) Agar (Oxoid, Hampshire). Presumptive large yellow colonies on TCBS were confirmed using API 20E strips (bioMérieux, France). Confirmation by serology was conducted using *Vibrio cholerae* O1 polyvalent antisera (Remel Europe, UK). DNA of the *V. cholerae* isolate was extracted using QIAamp DNA Mini Kit (Qiagen, Hilden) according to the manufacturer’s instructions. The DNA was then used for confirmation of *V. cholerae* species, identification of serogroup and biotype, and presence of virulence genes (*ctx A*, *ctx B*) by PCR, using published primers, as listed in Table 2. The composition of the master-mix included 1X Q5 reaction buffer (New England Biolabs, Massachusetts), 200 μM of dNTPs (1^st^ BASE, Singapore), 1U of Q5 Hot Start High Fidelity DNA polymerase (New England Biolabs, Massachusetts), forward and reverse primers (Integrated DNA Technologies, Singapore) with concentrations listed in Table 2, 5 μl of the sample DNA, and nuclease free water to attain a final volume of 50 μl. Amplification was performed in a thermal cycler (ABI Systems GeneAmp PCR system 9700) with the following temperature ramping: 98 °C for 30 s, followed by 35 cycles of 98 °C for 10 s, 60 °C for 30 s, 72 °C for 30 s, and finally 72 °C for 10 min. All PCR amplified product sizes were analysed using QIAxcel DNA high resolution kit (Qiagen, Hilden).

### Isolation of *Aeromonas* spp.

When *Aeromonas* spp. were detected in faecal samples by the Seeplex Diarrhoea ACE system, 1 g portions were each suspended into 9 ml of Universal Pre-enrichment Broth (Acumedia, Michigan) and Alkaline Peptone Water (FDA-BAM media formulation) [11]. The broths were incubated at 37 °C for 24 h. A 10 μl loopful of each enriched broth was streaked onto SA agar (Himedia, India) and incubated at 30 °C for 24 h. Starch hydrolysis was observed by flooding each incubated plate with 5 ml of iodine solution (Oxoid, Hampshire). Presumptive yellow colonies with zones of clearing were confirmed using API 20E strips (bioMérieux, France).

### Isolation of *Shigella* spp.

When *Shigella* spp. were detected by the Seeplex Diarrhoea ACE system, 1 g portions of the faecal samples were suspended into 9 ml of *Shigella* broth (FDA-BAM media formulation) [12]. Isolation and confirmation were conducted based on procedures described in FDA-BAM Chapter 6 [13].

### Isolation of verocytotoxigenic *E. coli* (VTEC)

When VTECs were detected by the Seeplex Diarrhoea ACE system, 1 g portions of the faecal samples were added into 9 ml of the Universal Pre-enrichment Broth (Acumedia, Michigan) and incubated at 37 °C for 24 h. A 10 μl loopful of the enriched broth was sub-cultured onto Eosin Methylene Blue, Levine agar (Acumedia, Michigan) and incubated at 37 °C for 24 h. Blue black colonies were confirmed as *E. coli* based on positive indole test reactions (Remel, Kansas). *E. coli* colonies were then screened for the presence of vtx 1 and/or vtx 2 genes using DEC PCR kit (Statens Serum Institut, Hillerød) according to the manufacturer’s instructions.

### Statistical analysis

The percentage of specimens positive for at least one of the 18 organisms tested for, as well as the percentage of specimens positive for each organism (inclusive of samples with co-detection of pathogens) were determined. Where specimens were positive for two or more organisms, the percentage of co-detection for each combination of organisms was determined.

## Results

Thirty-two percent (32 %) of the faecal samples contained at least one of the 18 organisms screened for. Bacterial pathogens were detected in 21 % of the samples, while viral and parasitic pathogens were detected in 14 and 2 % of the samples respectively. A total of eight samples (8 %) were positive for more than one pathogen. The frequency of co-detection is shown in Table 3.

**Table 3:** Co-detection of targeted organisms in faecal samples (*n* = 100)

Pathogens co-detected in faecal samples	% faecal samples
*Aeromonas* spp.*, C. perfringens*	2
*Aeromonas* spp., astrovirus	1
*Campylobacter* spp.*,* norovirus GII	1
*Campylobacter* spp.*,* group A rotavirus	1
*Aeromonas* spp.*, Salmonella* spp., astrovirus	1
*Aeromonas* spp., *Campylobacter* spp., *E. coli* O157:H7	1
*Campylobacter* spp., *E. coli* O157:H7, verocytotoxigenic *E. coli*, norovirus GII	1

Table 1 shows the percentage of samples positive for each organism tested. The most frequently encountered organism was norovirus genogroup II (11 %), followed by *Aeromonas* spp. (9 %) and *Campylobacter* spp. (5 %). One sample (1 %) was PCR-positive for both VTEC and *E. coli* O157:H7.

The Seeplex Diarrhoea ACE system and the in-house singleplex PCR showed consistent results for the screening of *Salmonella* spp.. However, the Seeplex Diarrhoea ACE system was unable to detect *Campylobacter* spp. in five samples that tested positive using the in-house singleplex PCR. This may be due to the fact that Seeplex is designed to detect only *C. jejuni* and *C. coli* whereas the singleplex PCR is meant to target the *Campylobacter* genus. While *Aeromonas* spp. was detected in nine samples using the Seeplex Diarrhoea ACE system, only five samples were detected using the in-house singleplex PCR. This could be because Seeplex is designed to detect *A. bivalvium , A. hydrophila , A. media, A. salmonicida, A. sobria,* and *A. veronii* whereas the singleplex primers were designed based on the 16S rRNA gene region of *A. hydrophila* ATCC 7966 [14]*.*

One *Salmonella* serogroup C and one *Vibrio cholerae* O1 El Tor were successfully recovered from PCR-positive samples. The *V. cholerae* isolate was found to be positive for *ctxA* and *ctxB* virulence genes, which affirm its genetic potential to produce cholera toxin [15], a protein responsible for causing profuse watery diarrhoea and vomiting [16].

## Discussion

Due to the ease of access to healthcare facilities by the general public, acute gastroenteritis (AGE) is seldom life-threatening in Singapore. However, the disease burden could be further reduced by strategic control measures. National statistics obtained from foodborne outbreak investigations from 2009 to 2011 [17] revealed that 51.2 % of the faecal samples obtained from cases contained pathogens, 75 % of these being *Salmonella* spp.. Separately, a study conducted at a women’s and children’s hospital in Singapore [18] revealed that AGE represented 1.7 to 2.4 % of paediatric admissions in 2003 and 2004, involving mainly children below 4 years old. The study reported that 81.4 % of the acute diarrhoea cases were due to viral infections while 17.9 % were attributed to bacterial infections, predominately due to *Salmonella* spp.. While that study provided valuable insights into the aetiology of AGE in children, there is still insufficient understanding of the aetiological agents contributing to non-outbreak-related adult AGE cases in Singapore. This may be due to a number of factors, such as the fact that hospitals do not routinely screen for a wide range of diarrheagenic pathogens, or that primary healthcare providers seldom submit faecal samples from diarrhoeal patients in the community for laboratory analysis.

In order to design a more comprehensive study to understand the epidemiology of AGE in Singapore, we have conducted this pilot study to obtain some preliminary data on the frequency and distribution of pathogens in adults’ faecal samples collected from a hospital in Singapore. Our findings suggest that pathogens causing non-notifiable disease might contribute significantly to adult hospitalised AGE cases. However, as the samples analysed were from a hospital, the data obtained may not be representative of the country. With reference to other high-income countries, the positivity rate of pathogens determined in our study (32 %) is comparable with that in Australia, the U.S. and the U.K., but much lower than that in Germany [19–22]. In the U.S., it was estimated in 2011 that 44 % of the hospitalised cases of all age groups due to domestically acquired foodborne illnesses were caused by known pathogens [22], predominately due to non-typhoidal *Salmonella* and norovirus [23]. In Australia, approximately 25 % of the hospital diagnosed gastroenteritis cases of all age groups were attributed to known aetiological agents, in which *Campylobacter* and non-typhoidal *Salmonella* were identified as the leading causes [20]. In the U.K., pathogens were detected in 50 % of patients of all age groups with infectious intestinal diseases who presented to general practitioners (GP) [19]. In contrast, a higher positivity rate of 82 % was detected in hospitalised adults suffering from AGE in Germany [21]. The variations in the positivity rates among studies should however be interpreted with caution as they could be due to numerous factors such as differences in case definitions used, screening panels, assay methods, nature of AGE cases (community vs. hospital), dietary factors and demographic profiles (age in particular) among countries. Additionally, it is noteworthy that as diarrhoea is a non-specific symptom observed in many diseases, it is difficult to rule out diarrhoeal cases caused by non-infectious diseases without obtaining epidemiological information and a medical history.

The predominant pathogen detected in our study was norovirus genogroup II (11 %). Norovirus is the leading cause of acute gastroenteritis (AGE) in many high-income countries such as the U.S., Australia and the U.K. [4, 20, 24]. Norovirus infection is highly contagious and can be transmitted through multiple routes, including through the ingestion of naturally contaminated food (such as salads, shellfish, water), or through person-to-person (e.g. aerosolised vomit) or environmental transmission (e.g. fomites) [25–30]. The positivity rate obtained in our study (11 %) is comparable with that of other studies conducted on AGE cases of all age groups in the U.K., Hong Kong S.A.R. and Taiwan [19, 30, 31]. From a global perspective, norovirus genogroup II genotype 4 (GII.4) is assessed to be responsible for most outbreaks and community cases of AGE and has been associated with an increased rate of hospitalisation and death during outbreaks based on data published between 1993 and 2011 [32]. Currently, screening of norovirus in adult diarrhoeal patients is not routinely performed in Singapore. Because there is no medical treatment for norovirus infection, the screening of norovirus may only offer limited benefits in clinical management. Nevertheless, it may be of public health value to combine epidemiological studies with further molecular characterisation of local norovirus strains, obtained from clinical and environmental sources, to determine the predominant modes of transmission. This may allow the design of relevant control measures and reduce the associated disease burden. Future work could also include the quantification of faecal viral loads using real-time reverse transcriptase PCR (RT-PCR). Indeed, as real time RT-PCR is able to detect low concentrations of norovirus, previous studies suggested the use of a suitable Cycle threshold (Ct) value as a cut-off point to distinguish between asymptomatic norovirus carriage and norovirus infection based on Receiver-Operating Characteristics (ROC) analysis [33]. This would in turn help to guide operational efforts by public health agencies to control and minimise the societal burden of AGE caused by norovirus.

The prevalence of *Aeromonas* spp. (9 %) in this study was higher than that of other bacteria. *Aeromonas* spp.*,* are widely distributed in the aquatic environment and have been isolated from many food types including meat, poultry, fish, seafood and vegetables [34]. *A. hydrophila, A. caviae* and *A. veronii,* in particular, are suspected of being emerging pathogens as they are capable of producing virulence factors even under cold temperatures [34] and may pose a potential hazard in chilled ready-to-eat food. However, their detection in clinical samples should be interpreted with caution as it may not always be associated with disease. A study conducted in Brazil showed a significant difference in the prevalence of *Aeromonas* spp. observed between out-patient diarrhoeal cases and healthy controls [35]. However, another study conducted in the U.K. cited that the detection rates of *Aeromonas* spp. in diarrhoeal patients (presented to community practitioners) and asymptomatic controls were similar [36]. Unfortunately, as healthy controls were not included in our study, and as the recovery of *Aeromonas* isolates from the frozen samples for speciation and virotyping was unsuccessful, the actual contribution of *Aeromonas* spp. to AGE symptoms cannot be ascertained further.

Verotoxigenic *E. coli* (VTEC), also known as Shiga toxin-producing *E. coli* (STEC), refers to a group of diarrheagenic *E. coli* which produces stx 1 and/or stx 2 toxins [37]. Enterohaemorrhagic *E. coli* (EHEC), a virotype of VTEC, may cause illness in humans [38]. As EHEC may cause bloody diarrhoea and potentially haemolytic uremic syndrome in severe cases [37], it is not surprising to detect their presence in hospitalised cases. In our study, one faecal sample was PCR-positive for both VTEC and *E. coli* O157:H7 although it was not detected or reported by the diagnostic laboratory. *E. coli* O157:H7 is a serotype which has frequently been encountered in clinical cases worldwide [37]. Other EHEC serotypes known to be associated with foodborne diseases include O26, O45, O91, O103, O104, O111, O121 and O145 [38]. In 2011, serotype O104:H4 caused a major outbreak in Germany due to its ability to express virulence characteristics of both enteroaggregative *E. coli* (EAEC) and EHEC [39]. Foods implicated in past EHEC outbreaks include ground meat, unpasteurised apple juices, raw milk, fermented hard salami and sprouted seeds [37, 40]. Environmental transmission of EHEC via contact with manure, animals and infected people has also been documented [41]. Although the detection of VTEC and *E. coli* O157:H7 in our samples may be of concern, it is noteworthy that no EHEC-related outbreak has ever been reported in Singapore, and that the possibility of an imported sporadic case cannot be ruled out.

The prevalence of *Campylobacter* spp. (5 %) and *Salmonella* spp. (1 %) in this study was relatively low in comparison to that of other targeted organisms. In several high-income countries, both *Campylobacter* spp. and non-typhoidal *Salmonella* spp. are determined to be the leading causes for acute gastroenteritis (AGE) [20, 21, 23, 24, 42]. While hospitalisation due to campylobacteriosis is generally less common than that due to salmonellosis in the U.S. and the U.K., campylobacteriosis is more frequently encountered than salmonellosis among AGE hospitalised cases in Germany [21, 24, 42]. In the U.S., *Campylobacter* and *Salmonella* are estimated to be responsible for 15 and 35 % of hospitalisations due to foodborne illness respectively (all age groups) [24]. In the U.K., it was estimated that about 9 to 18 % of foodborne hospitalisations of all age groups were due to *Campylobacter* and about 33 to 48 % were due to *Salmonella* [42]. In Germany, 35 and 20 % of the AGE hospitalised cases were due to *Campylobacter* and *Salmonella* respectively [21]. The low identification rate of *Salmonella* spp. in this study suggests that this pathogen may not be a major cause of hospitalisation in adults. Globally, salmonellosis has been associated with the consumption of contaminated food such as meat, eggs, poultry, fish, peanut butter and spices, as well as contact with pets such as turtles, frogs and chicks [43]. Campylobacteriosis, on the other hand, has generally been linked to animal contact, cross-contamination of food, as well as the consumption of raw milk, unpasteurised cheese, and undercooked poultry [44]. As *Salmonella* and *Campylobacter* can result in severe complications and sometimes death among the vulnerable populations such as the young and elderly [43, 44], continuous vigilance in the form of food safety education and surveillance efforts is required in order to safeguard public health.

The *Vibrio cholerae* isolate recovered in this study was found to be of serogroup O1 and biotype El Tor. Worldwide, *V. cholerae* serogroup O1 and serogroup O139 are recognised as the cause for cholera outbreaks [45]. The O1 serogroup consists of two biotypes, namely classical and El Tor [45]. The classical biotype was associated with pandemics prior to 1961 but was overtaken by the El Tor biotype thereafter [45]. Cholera is commonly linked to the consumption of contaminated water, ice and food, in particular seafood [46]. Between 2009 and 2013, the incidence of cholera in Singapore was between 0.02 and 0.08 per 100,000 population [5, 6, 47–49]. More than half of these cases were imported and *V. cholerae* serogroup O1 was identified as the most common serogroup responsible for cholera in Singapore [5, 6, 47–49]. Although the detection of *Vibrio cholerae* O1 El Tor may suggest a public health concern, it is worth mentioning that the incidence of cholera has always been low in Singapore. This is possibly due to the accessibility of potable water and sanitation infrastructures by the general public. Nevertheless, public health agencies should continue to monitor for signs of outbreaks and strive to maintain high standards of public hygiene and cleanliness.

It is noteworthy that there are several limitations in this preliminary study. The sample size was small and the samples were collected from a public hospital which may not be representative of the country. Due to the absence of medical history and epidemiological data from patients, it is difficult to determine the likely risk exposures and factors associated to these AGE cases, as well as to rule out cases of non-infectious diarrhoea. The lack of pathogen prevalence data from a healthy control group also makes it hard to assess whether the high prevalence of certain pathogens such as *Aeromonas* spp. were clinically significant. Furthermore, as adults are seldom admitted to hospitals for the treatment of diarrhoea in Singapore, the faecal samples obtained in this study might not truly reflect important aetiological agents affecting the community. Finally, the panel of pathogens targeted in our study does not represent an exhaustive list of pathogens that may be of public health interest. For instance, the screening of sapovirus, an important calicivirus also known to cause AGE, was not conducted in this study. The detection rates of sapovirus in stools were reported to reach levels as high as 9.2 % in specimens collected from a community cohort in the United Kingdom [19]. Such findings suggest that the contribution of sapovirus to disease burden may be higher than previously thought. Although no local data are available on its importance in Singapore, sapovirus, which was previously known to affect mostly children, has recently increasingly been reported to be associated with infections and outbreaks among adults in other countries, including in Asia [4, 19, 50–53]. Thus, the contribution of sapovirus to the disease burden of AGE in Singapore may need to be investigated further in future studies.

## Conclusion

Our preliminary findings suggest that pathogens causing non-notifiable foodborne diseases, such as norovirus, may contribute considerably to adult hospital attendances. However, as the samples analysed were obtained from a hospital, the data obtained may not be representative of the country as a whole. In order to better understand the epidemiology of AGE in Singapore, a larger study involving the analysis of faecal samples and risk exposure data collected from symptomatic and asymptomatic individuals in non-hospital settings may be beneficial to better characterise the epidemiology and transmission routes of AGE pathogens circulating in the community, and to better define priorities for their control. The screening of sapovirus and other pathogenic *E. coli,* such as enterotoxigenic *E. coli,* enteropathogenic *E. coli,* enteroaggregative *E. coli,* enteroinvasive *E. coli* and diffusely adherent *E. coli* could also be considered for future studies.
